# Circulating and Cardiac Tissue miRNAs in Children with Dilated Cardiomyopathy

**DOI:** 10.3390/jcdd10090391

**Published:** 2023-09-11

**Authors:** Frehiwet T. Hailu, Anis Karimpour-Fard, Bonnie Neltner, Brian L. Stauffer, Steven Lipshultz, Shelley D. Miyamoto, Carmen C. Sucharov

**Affiliations:** 1 Department of Medicine, Division of Cardiology, University of Colorado School of Medicine, Aurora, CO 80045, USA; frehiwet.hailu@cuanschutz.edu (F.T.H.);; 2 Department of Biomedical informatics, University of Colorado, Aurora, CO 80045, USA; 3 Division of Cardiology, Denver Health and Hospital Authority, Denver, CO 80204, USA; 4 Department of Pediatrics, University at Buffalo Jacobs School of Medicine and Biomedical Sciences, Oishei Children’s Hospital, Buffalo, NY 14203, USA; 5 Department of Pediatrics, University of Colorado School of Medicine, Children’s Hospital Colorado, Aurora, CO 80045, USA

**Keywords:** miRNA, heart failure, pediatrics, tissue, serum

## Abstract

microRNAs (miRs) are small non-coding single-stranded RNAs that regulate gene expression. We previously evaluated expression of miRs in the cardiac tissue of children with dilated cardiomyopathy (DCM) using miRNA-seq. However, a comparative analysis of serum and cardiac miRs has not been performed in this population. The current study aimed to evaluate miR levels in the serum of pediatric DCM patients compared to healthy non-failing (NF) donor controls and investigate the association between miR levels in tissue and sera from the same pediatric DCM patients. Defining the relationship between serum and tissue miRs may allow the use of circulating miRs as surrogate markers of cardiac miRs. miR levels were investigated through miR-array in sera [n = 10 NF, n = 12 DCM] and miR-seq in tissue (n = 10 NF, n = 12 DCM). Pathway analysis was investigated using the miR enrichment analysis and annotation tool (miEAA) for the five miRs commonly dysregulated in the sera and tissue of pediatric DCM patients. Functional analysis of miRs commonly dysregulated in the sera and tissue of pediatric DCM patients suggests altered pathways related to cell growth, differentiation and proliferation, inflammation, mitochondrial function, and metabolism. These findings suggest that circulating miRs could reflect altered levels of cardiac tissue miRs.

## 1. Introduction

Dilated cardiomyopathy (DCM) is the most common form of cardiomyopathy, the most frequent cause of heart failure (HF), and the most common reason for cardiac transplantation in adults and children [1]. Several studies in adults with HF have led to substantial improvements in the diagnosis, treatment, and prognosis for these patients [2]. However, therapeutic practices in children with DCM are based on adult patient guidelines, but therapies effective in adults provide only modest improvement in children [3]. A retrospective observational study has reported that there is some improvement in transplant-free survival in children with DCM [4]. However, the rate of transplantation in these patients has not changed, and nearly 50% of children with DCM die or require cardiac transplant within 5 years of diagnosis [4,5], indicating a critical need for new approaches to treat these children.

We previously identified unique molecular characteristics in the hearts of children with DCM [6,7,8,9], including unique age-dependent differences in microRNA (miR) expression [10,11]. miRs are small non-coding RNAs that regulate gene expression through seed sequence recognition of the 3′ untranslated region (UTR) of messenger RNAs [12]. miRs represent a potential novel therapeutic target for several cardiovascular disorders including HF [13]. miRs are stable in the circulation and are potential biomarkers for diagnosis, prognosis, and response to therapy in HF [14].

In our prior study, we identified specific miRs that are differentially regulated between children who needed heart transplants and those who recovered from HF, which increases the potential of using circulating miRs as a biomarker of recovery in children with DCM [15]. Although there is increasing interest in circulating miRs in HF, their origin and function are not completely understood [14]. Moreover, it is not clear whether miRs circulating in the sera of children with DCM can accurately represent miR expression patterns in heart tissue. This gap in knowledge limits the understanding of the biological significance of altered serum miR levels. Investigating whether circulating miR levels reflect cardiac tissue miR expression is important to understanding the potential biological significances of miRs detected in the sera.

In the current study, we evaluated the expression profile of miRs in the sera of children with DCM and age-matched non-failing (NF) controls. Using our published miR-seq data [11], we compared levels of miRs differentially regulated in sera to miRs dysregulated in heart tissue. Furthermore, the miR enrichment analysis and annotation tool predicted putative signaling pathways related to the differentially expressed circulating miRs, including miRs that are similarly dysregulated in sera and cardiac tissue, and identified pathways related to cell growth, differentiation and proliferation, inflammation, mitochondrial function, and metabolism.

## 2. Materials and Methods

### 2.1. Human Samples

Human participants were males and females of all races and ethnic backgrounds <18 years (n = 10 NF and n = 12 DCM) who gave informed consent and donated their hearts or blood to the institutional review board-approved Investigations of Pediatric Heart Disease study at the University of Colorado, Anschutz Medical Campus. DCM patient blood was collected on the day of heart transplant at Children’s Hospital Colorado. Left ventricular (LV) tissue from children with HF secondary to DCM was collected in the operating room at the time of heart transplantation. Inclusion criteria were age <18 years and diagnosis of DCM defined as ejection fraction <50% or fractional shortening <25% and/or a dilated left ventricle (left ventricle end-diastolic volume *z*-score ≥ 2). Patients with a primary diagnosis of congenital heart disease were excluded.

Blood from NF controls was collected from age-matched volunteers with normal heart structure and function. Non-failing LV tissue was collected from organ donors with normal heart structure and function, whose hearts could not be placed for technical reasons (size or blood-type mismatch). At the time of cardiac transplantation or donation, the LV was rapidly dissected in the operating room, flash-frozen, and stored at −80 °C until further use. Descriptive details for all study participants are listed in Table S1.

### 2.2. miR Array and RNA-Seq

miR array was performed using TaqMan Open Array miR panel (Life Technologies, Carlsbad, CA, USA), which can identify 381 RNAs from plasma or serum. Experiments were performed according to manufacturer’s recommendation, with modifications essentially as previously described [16,17]. One 384-well plate was used for each sample to detect 380 miRs and U6 in four wells. We compared the expression profile of miRs in the LV tissue of children with DCM versus non-failing pediatric controls using miR seq [11].

### 2.3. Array Analysis

Expression Suite Software version 1.1 (Life Technologies, Carlsbad, CA, USA) was used to perform array analysis. Among more than 400 pediatric samples from different etiologies and healthy controls (data not shown), we have identified miR-320 as the least variable circulating miR, and so it was used as an internal control. Data were analyzed with the Wilcoxon signed-rank test, and only q values < 0.05 were considered to be statistically significant. Unsupervised classification with random forest (RF) analysis was performed in R (https://cran.r-project.org/web/packages/randomForest/index.html (accessed on 28 June 2023)) using 50,000 trees to identify the top three miRs that differentiated between patients and controls. RF algorithms measure the importance of a variable or how much it contributes to the predictive accuracy through two different methods (mean decrease accuracy and mean decrease Gini Index). Sensitivity and specificity were calculated from a receiver operating curve (ROC) for the three miRs providing the best differentiation. A pROC package was used to calculate the area under the operating characteristic curve (AUC). Heatmaps were plotted with the heatmap2 function in gplots package in R.

### 2.4. Pathway Analysis

To identify putative pathways or targets of miR, the miR enrichment analysis and annotation tool (miEAA) was applied to detect KEGG pathways. Pathway analyses were performed using miRs with the same directional dysregulation in the sera and cardiac tissue, and for miRs exclusively dysregulated in the sera. A q-value < 0.05 was considered statistically significant.

### 2.5. Statistical Methods

The significance of the overlap between significantly dysregulated tissue and circulating miRs was evaluated with Pearson’s correlation and the chi-square test. The total number of genes used in chi-square calculations was based on 123 miRs identified in the arrays. Chi-square was calculated based on the overlap between significantly dysregulated miRs. Logistic regression was performed on the top eight miRs using R, and *p*-values were adjusted, and q < 0.05 was considered significant.

## 3. Results

### 3.1. Study Participant Characteristics

Characteristics of the study participants are listed in Table S1. The median age for pediatric NF donors was 7.2 years with an interquartile range (IQR) of 3.5–10.2 years, and a median age of 7.2 years with an IQR of 3.7–11.6 years for children with DCM. Of the study participants, 50% of the pediatric NF donors and 58% of the children with DCM were females. Phosphodiesterase 3 inhibitor (PDE3i), angiotensin converting enzyme inhibitor, beta blockers, and digoxin were more commonly used in the children with DCM.

### 3.2. Identification of Differentially Expressed Circulating miRs

**Figure 1:**
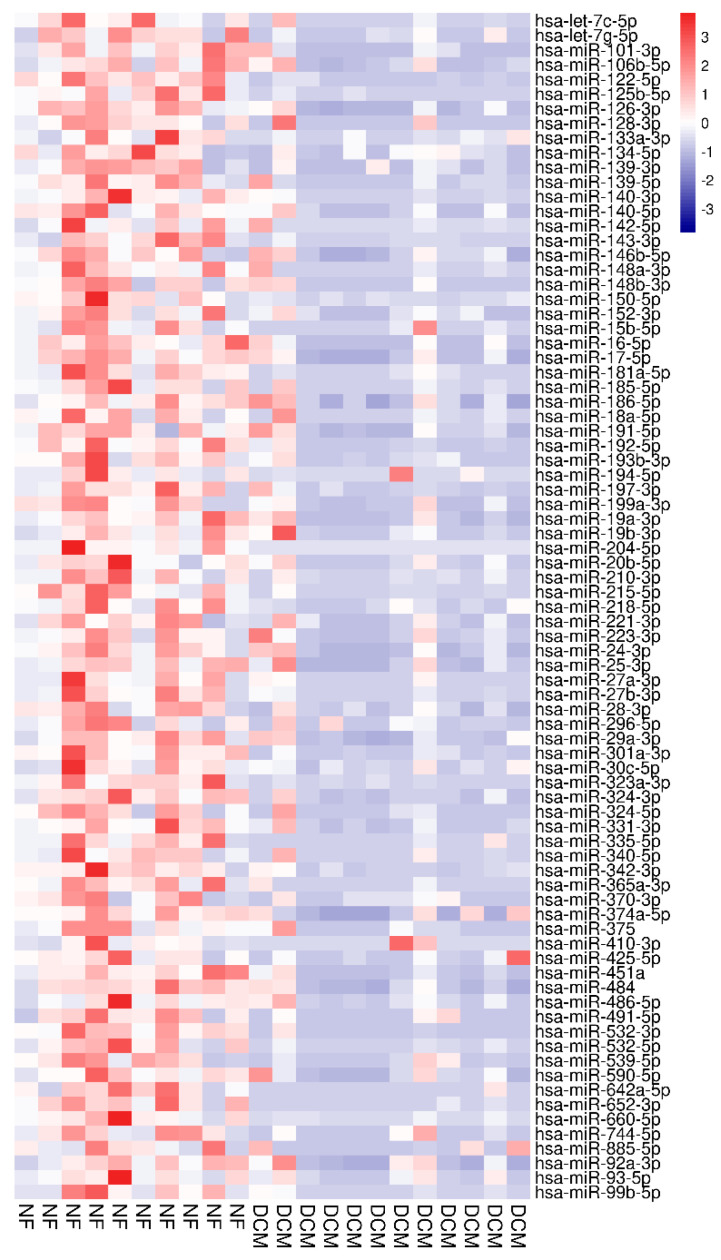
Heat map based on *t*-test from arrays separating NF controls (red labels, n = 10) from DCM subjects (blue labels, n = 12). Red indicates upregulation; blue indicates down-regulation. miRNAs in the right hand column are ranked by *t*-test q-value.

miR arrays identified a total of 123 circulating miRs. A total of 81 miRs were differentially expressed between the two groups q < 0.05 (Figure 1 and Table 1).

**Figure 2:**
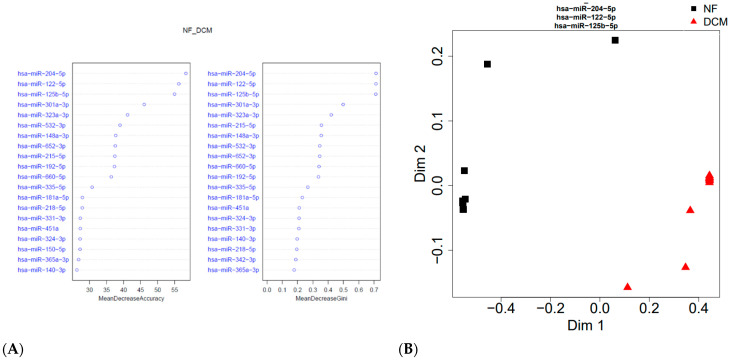

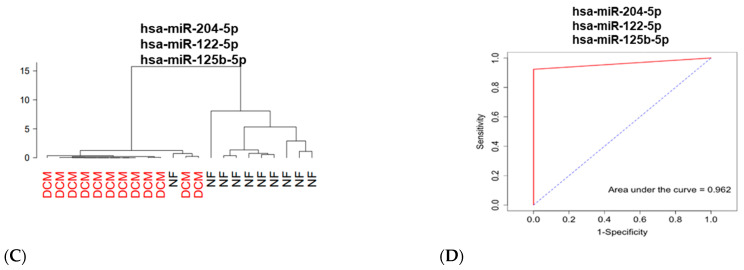
Serum miRNAs differentiated pediatric DCM subjects (n = 12) from age-matched NF controls (n = 10). (**A**) Rank of most important miRNAs using multidimensional scaling. (**B**) Random forest (RF) analysis demonstrated that miR-204-5p, miR-125b-5p, and miR-122-5p differentiated the two groups. (**C**) Hierarchal clustering showed separation between the DCM and NF controls. (**D**) Receiver operating curves using the top three miRNAs showed an area under the curve of 0.96.

The expression of all differentially regulated miRs was down-regulated in the sera of DCM patients compared to the NF control group. The rank of the most important miRs, as determined by RF multidimensional scaling of the estimated proximity matrix plots, is displayed in Figure 2A. The three miRs providing the best differentiation between DCM and NF controls were miR-204-5p, miR-125b-5p, and miR-122-5p. RF analysis using these miRs demonstrated a stark differentiation between groups (Figure 2B). Hierarchal clustering effectively separated the groups using these miRs (Figure 2C). ROC was generated based on miR-204-5p, miR-125b-5p, and miR-122-5p and had 96% sensitivity and specificity to distinguish children with DCM from NF controls (Figure 2D).

**Figure 3:**
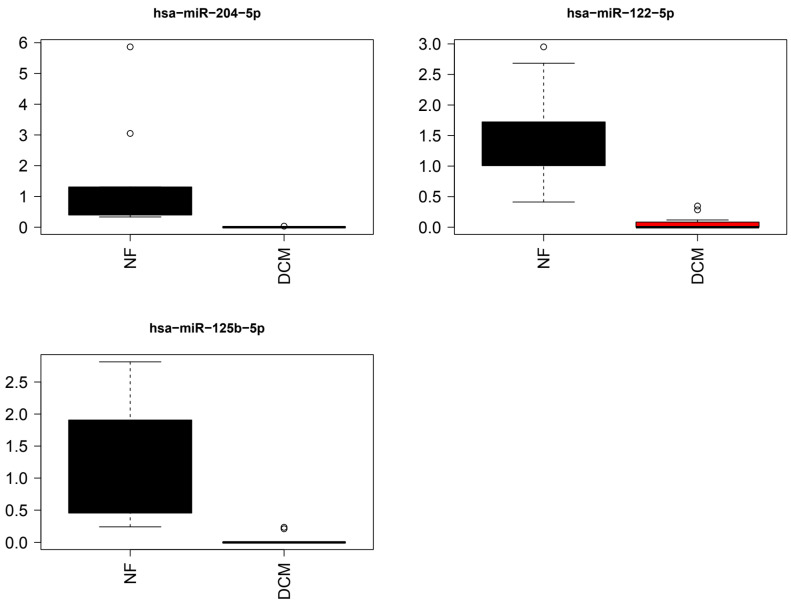
Bar graphs of the top three differentially expressed miRNAs from miRNA arrays. Individual miRNAs selected based on *p* < 0.0001. Relative miRNA expression of select miRNAs among DCM subjects n = 12 and NF controls n = 10. The values that are more than 1.5 times interquartile range away from the box are shown as circles.

Differences in the expression of miR-204-5p, miR-125b-5p, and miR-122-5p between DCM and NF controls were statistically significant (q < 0.05) (Figure 3).

### 3.3. Association between Circulating and Cardiac Tissue miRs

We compared the expression profile of differentially regulated circulating miRs with data from our RNA-seq investigations of cardiac miRs [11]. The comparison of DCM miRs was performed using serum and tissue from the same patient obtained on the day of heart transplant. Serum and tissue from NF controls were matched for age and sex but were not from the same subject. Three miRs (miR-204-5p, miR-125b-5p, miR-301a-3p) were significantly up-regulated in DCM tissue and down-regulated in DCM sera compared to NF controls (Table 2). Five miRs (miR-133a-3p, miR-150-5p, miR-486-5p, miR-17-5p, miR-92a-3p) were significantly down-regulated in both the tissue and sera of DCM patients compared to NF controls. In addition, 73 miRs were uniquely down-regulated in the sera of DCM patients compared to the NF controls, and 10 other miRs (4 up-regulated and 6 down-regulated) were differentially regulated only in DCM tissue compared to the NF group (Table 2).

We next sought to determine if the overlap between the miRs that change in sera and tissue was significant. We performed Pearson’s correlations between the tissue and sera samples. The chi-square test was performed on miRs that had significant correlation. Eight miRs were dysregulated in both cardiac tissue and serum (three miRs dysregulated in the opposite direction, and five miRs down-regulated in both compartment), which resulted in *p* = 0.016, suggesting that the overlap between tissue and circulating miRs is greater than expected by chance. We also performed logistic regression of these top eight significantly dysregulated circulating and tissue miRs to determine if there is a correlation between the miRs and the two compartments that was significant. As shown in Table 3, these top eight tissue miRs are significant by logistic regression, suggesting dependency on the regulation of tissue and circulating miRs.

### 3.4. Pathway Analysis

Pathway analysis of the 73 miRs that were down-regulated in the sera of children with DCM compared to NF controls demonstrated over-representation of pathways involved in cell growth, proliferation and differentiation (cAMP signaling pathway, signaling pathways regulating pluripotency of stem cells, and TGF-beta signaling pathway), inflammation (toll-like receptor pathway and chemokine signaling pathway), cardiac muscle contraction, cardiomyopathy (hypertrophic cardiomyopathy, arrhythmogenic right ventricular cardiomyopathy, and dilated cardiomyopathy), metabolism (citrate cycle TCA cycle, ratty acid biosynthesis, pyruvate metabolism, and glucagon signaling pathway), mitochondrial function (oxidative phosphorylation) and activation of mitophagy, autophagy, and ubiquitin-mediated proteolysis (Table 4). All putative pathways are depicted in Table S2.

Similarly, pathway analysis of the five miRs down-regulated in both the sera and tissue of the DCM group compared to NF controls as well as that of the three miRs up-regulated in tissue and down-regulated in the sera of the DCM group compared to NF controls indicated over-representation of pathways involved mainly in metabolism (inositol phosphate metabolism, carbohydrate digestion and absorption, propanoate metabolism, fructose and mannose metabolism, and pyruvate metabolism, citrate cycle TCA cycle, valine, leucine, and isoleucine degradation, and ratty acid biosynthesis) (Table 5).

## 4. Discussion

Our prior studies demonstrated the potential for circulating miRs to predict the need for transplantation or recovery of heart function in children with DCM [15]. However, the association between the profiles of pediatric DCM serum and cardiac tissue miRs has not been investigated. In this study, we identified, by miR array, 81 miRs significantly dysregulated in the sera of pediatric DCM patients compared to NF controls. In contrast to adults with congestive heart failure [18], all differentially expressed circulating miRs in children with DCM were down-regulated compared to NF controls, which underscores the importance of conducting pediatric-focused studies. Furthermore, using our published miR-seq data [11], we evaluated miR expression in serum and myocardial (LV) tissue samples from the same pediatric DCM patients and from age-matched NF controls (NF samples not paired) and determined that the correlation between dysregulated miRs in sera and cardiac tissue is significant, suggesting circulating miRs can reflect cardiac tissue levels. This investigation highlights the importance of pursuing blood-based miRs as a non-invasive approach to better understand the biological significance of miRs in children with heart failure.

It has been reported that miRs are electively released from cardiomyocytes in response to stress or injury, providing great potential as a cardiac biomarker [19]. We identified several miRs that are down-regulated in both the sera and tissue of children with DCM, suggesting circulating levels reflect the lower cardiac levels. However, some miRs are increased in the tissue and decreased in the circulation of children with DCM, which could suggest selective retention of these miRs in cardiac tissue.

In addition to their intracellular activities, circulating miRs play a significant role mediating intercellular communication through their impact on proteins, lipids, or extracellular vesicle carriers. For example, circulating miRs secreted by donor cells can be delivered into recipient cells where they function as endogenous miRs, altering gene expression [20]. Moreover, previous studies have shown that miR-enriched extracellular vesicles can be secreted by immune cells functioning as a hormone-like effector in cardiovascular health and disease [21,22,23]. Previously, we have reported that the number of circulating exosomes in the serum of children with DCM is lower compared to NF controls [24], which supports our findings that all dysregulated circulating miRs between children with DCM and NF controls were down-regulated.

Several miRs were differentially expressed in the sera of children with DCM compared to NF controls, with the top three differentially expressed miRs being miR-204-5p, miR-125b-5p, and miR-122-5p. Consistent with our previous study in a separate cohort of children with DCM, all miRs differentially regulated in DCM sera were down-regulated [25]. A previous study showed that miR-204-5p can inhibit the development of cardiac hypertrophy and dysfunction [26], and, in agreement with our findings, lower serum levels of miR-204-5p are associated with the presence of cardiovascular disease [27]. Also consistent with our findings, miR-125b-5p has been shown to be down-regulated in the circulation of adults with end-stage DCM and ischemic cardiomyopathy [28,29]. This down-regulation has been associated with acute myocardial infarction in adults [30] and with increased cardiomyocyte apoptosis in mice [31]. miR-122 has been highly investigated in cardiovascular diseases. Several studies have shown that miR-122 regulates cardiovascular inflammation, autophagy, apoptosis, oxidative stress, fibrosis, and dysfunction and appears to be a direct participant in the development of cardiovascular diseases including heart failure [32,33,34]. Additionally, a recent study in rats showed that over-expression of miR-122-5p regulates Ang II-triggered increased apoptosis and reduced SIRT6, ELA, and ACE2 levels, which was alleviated by administration of miR-122-5p inhibitor [34]. We found lower levels of miR-122 in pediatric DCM serum compared to NF controls, which could indicate an attempt at compensating for HF. Interestingly, we also previously showed that miR-122-5p levels are decreased in the hearts of children with DCM [11]. Using the current subset of patients, we did observe a decrease in miR-122-5p levels by *p*-value, but not by q-value (data not shown).

The results of the pathway analyses in this study are consistent with several of our prior findings. We previously performed pathway analyses of putative miR–mRNA pairs in pediatric DCM myocardia, and as was found in this study, these analyses predicted alterations in pathways related to cell proliferation, differentiation, inflammation, and mitochondrial function [11]. Our transcriptome investigations of the pediatric heart suggested an association between altered gene expression and incomplete cell differentiation [8]. In this study, pathway analysis predictions of altered circulating miRs indicate an over-representation of pathways involving cell growth, proliferation, and differentiation. Lastly, and in agreement with our prior study on mitochondrial dysfunction in children with DCM [35], circulating miR expression patterns predict mitochondrial dysfunction [36]. Altogether, our results suggest that miRs dysregulated in sera of children with DCM might modulate biological and pathological pathways that contribute to the progression of heart failure.

## 5. Conclusions

We found alterations in the expression levels of several miRs in the sera of children with DCM compared to NF healthy controls. We also compared paired circulating and tissue expression profile of miRs in children with DCM to serum and tissue from age-matched, but non-paired NF controls. miRs dysregulated in pediatric DCM sera are involved in pathways related to cell growth, proliferation and differentiation, inflammation, cardiomyopathy, metabolism, mitochondrial function, and activation of mitophagy and autophagy. Furthermore, our analyses showed that dysregulation of miRs in the circulation and heart tissue may be interdependent. These investigations provide a framework for future studies aimed at understanding the relationship between circulating and cardiac tissue miRs in pediatric heart failure. Moreover, further studies on the implication of dysregulated miRs in the hearts and circulation of children with DCM may contribute to the development of potential age-specific miR-based diagnostics and therapeutics.

## 6. Limitations

There are limitations to this study. (1) We recognize the number of patients is small. However, pediatric DCM is rare, and this cohort of patients provided a unique opportunity to investigate the relationship between cardiac and circulating miRs. (2) We do not have the ability to investigate the relationship between circulating and cardiac miRs from the same NF subjects. Sex and age were matched to the best of our ability. (3) We have no ability to test if lower levels of circulating miRs are due to retention of these miRs in the heart or if they are released from other organs. (4) We recognize this is a hypothesis-generating study and that further studies will be necessary to define the cause and consequence of circulating miRs as they relate to cardiac function. (5) RNA seq requires approximately 200 μL of sera to detect miRs comparing to array, which is a substantial amount in the pediatric population. (6) Due to a small sample size and different drug permutations, it is not possible to test the effect of drugs on miR levels.

## Figures and Tables

**Table 1:** Microarray data on miRNAs differentially expressed in the sera of 10 children with heart failure and 12 healthy controls.

miRNA	Fold Change	qValue_wilcox
hsa-let-7c-5p	−0.89117	0.006447
hsa-let-7g-5p	−0.74433	0.011349
hsa-miR-101-3p	−0.84796	0.006447
hsa-miR-106b-5p	−0.59646	0.015417
hsa-miR-122-5p	−1.46702	0.002372
hsa-miR-125b-5p	−1.1267	0.002331
hsa-miR-126-3p	−0.66084	0.004093
hsa-miR-128-3p	−0.59713	0.020073
hsa-miR-133a-3p	−1.13195	0.048692
hsa-miR-134-5p	−0.63877	0.04314
hsa-miR-139-3p	−0.77407	0.011413
hsa-miR-139-5p	−0.70595	0.006682
hsa-miR-140-3p	−1.09951	0.002472
hsa-miR-140-5p	−0.77305	0.008129
hsa-miR-142-5p	−0.80908	0.00872
hsa-miR-143-3p	−1.16193	0.00387
hsa-miR-146b-5p	−0.38407	0.048692
hsa-miR-148a-3p	−1.0221	0.002982
hsa-miR-148b-3p	−0.7088	0.028505
hsa-miR-150-5p	−0.72873	0.002472
hsa-miR-152-3p	−0.91037	0.021286
hsa-miR-15b-5p	−0.74914	0.009937
hsa-miR-16-5p	−0.67087	0.004093
hsa-miR-17-5p	−0.63261	0.005181
hsa-miR-181a-5p	−0.96636	0.002372
hsa-miR-185-5p	−0.9488	0.009937
hsa-miR-186-5p	−0.40663	0.040325
hsa-miR-18a-5p	−0.66868	0.012857
hsa-miR-191-5p	−0.4202	0.046648
hsa-miR-192-5p	−0.84033	0.002472
hsa-miR-193b-3p	−0.87364	0.006447
hsa-miR-194-5p	−0.57466	0.012857
hsa-miR-197-3p	−0.79624	0.006447
hsa-miR-199a-3p	−0.75922	0.008129
hsa-miR-19a-3p	−0.6605	0.016026
hsa-miR-19b-3p	−0.47258	0.018154
hsa-miR-204-5p	−1.42388	0.002331
hsa-miR-20b-5p	−0.70575	0.034222
hsa-miR-210-3p	−1.05106	0.006447
hsa-miR-215-5p	−1.11726	0.002372
hsa-miR-218-5p	−1.05591	0.005706
hsa-miR-221-3p	−0.73779	0.029695
hsa-miR-223-3p	−0.56938	0.026463
hsa-miR-24-3p	−0.64909	0.012857
hsa-miR-25-3p	−0.62173	0.011006
hsa-miR-27a-3p	−1.06271	0.012857
hsa-miR-27b-3p	−1.28136	0.003618
hsa-miR-28-3p	−0.66936	0.009539
hsa-miR-296-5p	−0.73451	0.040325
hsa-miR-29a-3p	−0.53525	0.011349
hsa-miR-301a-3p	−0.9541	0.002372
hsa-miR-30c-5p	−0.77104	0.029695
hsa-miR-323a-3p	−1.05664	0.002372
hsa-miR-324-3p	−0.86761	0.002982
hsa-miR-324-5p	−0.69613	0.014925
hsa-miR-331-3p	−0.67157	0.00387
hsa-miR-335-5p	−1.18965	0.002372
hsa-miR-340-5p	−0.58158	0.015343
hsa-miR-342-3p	−0.5613	0.00369
hsa-miR-365a-3p	−1.25129	0.002787
hsa-miR-370-3p	−0.62826	0.044334
hsa-miR-374a-5p	−0.54854	0.038544
hsa-miR-375	−0.8206	0.008122
hsa-miR-410-3p	−0.48513	0.038751
hsa-miR-425-5p	−0.59274	0.016943
hsa-miR-451a	−0.80526	0.002372
hsa-miR-484	−0.56538	0.002372
hsa-miR-486-5p	−0.64054	0.038544
hsa-miR-491-5p	−0.98398	0.006447
hsa-miR-532-3p	−0.83425	0.002372
hsa-miR-532-5p	−1.29285	0.006447
hsa-miR-539-5p	−0.73027	0.012857
hsa-miR-590-5p	−0.6414	0.018154
hsa-miR-642a-5p	−0.62641	0.005706
hsa-miR-652-3p	−0.91256	0.002372
hsa-miR-660-5p	−1.06831	0.00387
hsa-miR-744-5p	−0.71785	0.009539
hsa-miR-885-5p	−0.90867	0.029695
hsa-miR-92a-3p	−0.49284	0.029695
hsa-miR-93-5p	−0.71524	0.028889
hsa-miR-99b-5p	−1.44717	0.008129

**Table 2:** Directionality of expression of miRNAs differentially regulated in the sera and heart tissue of children with DCM compared to non-failure controls.

miRNA	Increased in Sera	Decreased in Sera	Increased in Cardiac Tissue	Decreased in Cardiac Tissue
hsa-miR-204-5p		x	x	
hsa-miR-125b-5p		x	x	
hsa-miR-301a-3p		x	x	
hsa-miR-133a-3p		x		x
hsa-miR-150-5p		x		x
hsa-miR-486-5p		x		x
hsa-miR-17-5p		x		x
hsa-miR-92a-3p		x		x
hsa-miR-20a-5p				x
hsa-miR-212-3p				x
hsa-miR-132-3p				x
hsa-miR-193a-5p				x
hsa-miR-345-5p				x
hsa-let-7d-5p				x
hsa-miR-574-3p			x	
hsa-miR-376c-3p			x	
hsa-miR-195-5p			x	
hsa-miR-130b-3p			x	
hsa-miR-122-5p		x		
hsa-miR-99b-5p		x		
hsa-miR-532-5p		x		
hsa-miR-27b-3p		x		
hsa-miR-365a-3p		x		
hsa-miR-335-5p		x		
hsa-miR-143-3p		x		
hsa-miR-215-5p		x		
hsa-miR-140-3p		x		
hsa-miR-660-5p		x		
hsa-miR-27a-3p		x		
hsa-miR-323a-3p		x		
hsa-miR-218-5p		x		
hsa-miR-210-3p		x		
hsa-miR-148a-3p		x		
hsa-miR-491-5p		x		
hsa-miR-181a-5p		x		
hsa-miR-185-5p		x		
hsa-miR-652-3p		x		
hsa-miR-152-3p		x		
hsa-miR-885-5p		x		
hsa-let-7c-5p		x		
hsa-miR-193b-3p		x		
hsa-miR-324-3p		x		
hsa-miR-101-3p		x		
hsa-miR-192-5p		x		
hsa-miR-532-3p		x		
hsa-miR-375		x		
hsa-miR-142-5p		x		
hsa-miR-451a		x		
hsa-miR-197-3p		x		
hsa-miR-139-3p		x		
hsa-miR-140-5p		x		
hsa-miR-30c-5p		x		
hsa-miR-199a-3p		x		
hsa-miR-15b-5p		x		
hsa-let-7g-5p		x		
hsa-miR-221-3p		x		
hsa-miR-296-5p		x		
hsa-miR-539-5p		x		
hsa-miR-744-5p		x		
hsa-miR-93-5p		x		
hsa-miR-148b-3p		x		
hsa-miR-139-5p		x		
hsa-miR-20b-5p		x		
hsa-miR-324-5p		x		
hsa-miR-331-3p		x		
hsa-miR-16-5p		x		
hsa-miR-28-3p		x		
hsa-miR-18a-5p		x		
hsa-miR-126-3p		x		
hsa-miR-19a-3p		x		
hsa-miR-24-3p		x		
hsa-miR-590-5p		x		
hsa-miR-134-5p		x		
hsa-miR-370-3p		x		
hsa-miR-642a-5p		x		
hsa-miR-25-3p		x		
hsa-miR-128-3p		x		
hsa-miR-106b-5p		x		
hsa-miR-425-5p		x		
hsa-miR-340-5p		x		
hsa-miR-194-5p		x		
hsa-miR-223-3p		x		
hsa-miR-484		x		
hsa-miR-342-3p		x		
hsa-miR-374a-5p		x		
hsa-miR-29a-3p		x		
hsa-miR-410-3p		x		
hsa-miR-19b-3p		x		
hsa-miR-191-5p		x		
hsa-miR-186-5p		x		
hsa-miR-146b-5p		x		

**Table 3:** Logistic regression analysis of the top eight dysregulated circulating and tissue miRs.

miRNA	Coefficient	*p*-Value	q Value
hsa-miR-125b-5p	32.33467	0.006746	0.007709
hsa-133a-3p	−29.7126	0.004939	0.006585
hsa-miR-150-5p	−31.855	0.000407	0.001084
hsa-miR-17-5p	−36.6685	0.000131	0.000526
hsa-miR-204-5p	10.94161	0.002227	0.003563
hsa-miR-301a-3p	35.57683	0.000112	0.000526
hsa-miR-486-5p	−30.6944	0.000592	0.001184
hsa-miR-92a-3p	−31.5682	0.012177	0.012177

**Table 4:** Putative pathways affected by miRNAs exclusively down-regulated in the sera of children with DCM.

Pathway	*p*-Value
Adipocytokine signaling pathway	1.40 × 10^−12^
Mitophagy—animal	2.86 × 10^−12^
VEGF signaling pathway	1.38 × 10^−11^
Fatty acid biosynthesis	6.64 × 10^−11^
Toll-like receptor signaling pathway	6.73 × 10^−11^
IL-17 signaling pathway	7.41 × 10^−11^
Pyruvate metabolism	4.16 × 10^−10^
Glucagon signaling pathway	1.17 × 10^−9^
Carbohydrate digestion and absorption	1.99 × 10^−9^
Chemokine signaling pathway	4.82 × 10^−9^
Hedgehog signaling pathway	5.05 × 10^−9^
AMPK signaling pathway	1.44 × 10^−8^
Signaling pathways regulating pluripotency of stem cells	1.49 × 10^−8^
Wnt signaling pathway	2.37 × 10^−8^
Autophagy—other	4.16 × 10^−8^
Hippo signaling pathway	5.00 × 10^−8^
Fatty acid degradation	9.44 × 10^−8^
TGF-beta signaling pathway	1.00 × 10^−7^
Hypertrophic cardiomyopathy HCM	1.01 × 10^−7^
Glycolysis Gluconeogenesis	1.21 × 10^−7^
PPAR signaling pathway	1.41 × 10^−7^
Oxidative phosphorylation	2.45 × 10^−7^
TNF signaling pathway	3.92 × 10^−7^
Arrhythmogenic right ventricular cardiomyopathy ARVC	8.56 × 10^−7^
p53 signaling pathway	1.02 × 10^−6^
Ubiquitin mediated proteolysis	1.47 × 10^−6^
Cytokine–cytokine receptor interaction	2.85 × 10^−6^
Dilated cardiomyopathy DCM	3.15 × 10^−6^
FoxO signaling pathway	4.08 × 10^−6^
Hippo signaling pathway—multiple species	4.56 × 10^−6^
mTOR signaling pathway	1.06 × 10^−5^
Fat digestion and absorption	1.33 × 10^−5^
Citrate cycle TCA cycle	2.57 × 10^−5^
Autophagy—animal	2.71 × 10^−5^
Cardiac muscle contraction	3.04 × 10^−5^
Phosphatidylinositol signaling system	3.49 × 10^−5^
PI3K-Akt signaling pathway	4.11 × 10^−5^
MAPK signaling pathway	4.43 × 10^−5^
cAMP signaling pathway	4.51 × 10^−5^

**Table 5:** Putative pathways affected by miRNAs down-regulated in cardiac tissue and sera of children with DCM.

Pathway	*p*-Value
Fructose and mannose metabolism	0.0072082
Propanoate metabolism	0.0082026
Inositol phosphate metabolism	0.0100905
Carbohydrate digestion and absorption	0.0118522
Valine, leucine, and isoleucine degradation	0.0119277
VEGF signaling pathway	0.013063
Citrate cycle TCA cycle	0.015308
Arachidonic acid metabolism	0.0164423
Pyruvate metabolism	0.0165376
Fatty acid biosynthesis	0.0197426
Riboflavin metabolism	0.0218427
Pyrimidine metabolism	0.0247384
Glucagon signaling pathway	0.0401259

## Data Availability

The data presented in this study can be downloaded at: https://docs.google.com/spreadsheets/d/1UB41CIpS_REKE-tzQGRTlmEf0DBwc3Fuje3h_R91A3Q/edit?usp=sharing (accessed on 28 June 2023).

## Supplementary Materials

The following supporting information can be downloaded at: https://docs.google.com/document/d/1H7Ed-FaRkXPZDJ_8dVAO2VEWTfSG-in4/edit?usp=sharing&ouid=108956289605910293220&rtpof=true&sd=true, Table S1. Patient characteristics. ID = Identification, NF = Non-Failing, DCM = Dilated Cardiomyopathy, M = Male, F = Female, NA = Not Available, PDE3i = Phosphodiesterase3 Inhibitor, PDE5i = Phosphodiesterase5 Inhibitor, EP = Electrophysiology, SVT = Supraventricular tachycardia. Table S2. Putative pathways and the miRNAs exclusively down-regulated in the sera of children with DCM.
